# Neuroprotective and cognitive-enhancing effects of the combined extract of *Cyperus rotundus* and *Zingiber officinale*

**DOI:** 10.1186/s12906-017-1632-4

**Published:** 2017-03-03

**Authors:** Chatchada Sutalangka, Jintanaporn Wattanathorn

**Affiliations:** 1 Department of Physiology and Graduate School (Neuroscience Program), Faculty of Medicine, Khon Kaen University, Khon Kaen, 40002 Thailand; 2 Integrative Complementary Alternative Medicine Research and Development Center, Khon Kaen University, Khon Kaen, 40002 Thailand; 3 Department of Physiology, Faculty of Medicine, Khon Kaen University, Khon Kaen, 40002 Thailand

**Keywords:** *Cyperus rotundus*, *Zingiber officinale*, Dementia, Neurodegeneration

## Abstract

**Background:**

Currently, food supplements to improve age-related dementia are required. Therefore, we aimed to determine the effect of the combined extract of *Cyperus rotundus* and *Zingiber officinale* (CP1) on the improvement of age-related dementia in rats with AF64A-induced memory deficits.

**Methods:**

Male Wistar rats weighing 180-200 g were orally given CP1 at doses of 100, 200 and 300 mg.kg^-1^ BW for a period of 14 days after bilateral intracerebroventricular administration of AF64A. Spatial memory was assessed in all rats every 7 days throughout the 14 day-experimental period. At the end of the study, neuronal density, acetylcholinesterase (AChE) activity, oxidative stress status and the activation of MAPK cascades in the hippocampus were determined.

**Results:**

Enhanced memory, increased neuronal density, decreased AChE activity and decreased oxidative stress status together with activated pERK1/2 were observed in the hippocampus of CP1-treated rats. These results suggested that CP1 might improve memory via enhanced cholinergic function and decreased neurodegeneration and oxidative stress.

**Conclusions:**

CP1 is a potential novel food supplement for dementia. However, further investigations on the subchronic toxicity of CP1 and drug interactions are required.

**Electronic supplementary material:**

The online version of this article (doi:10.1186/s12906-017-1632-4) contains supplementary material, which is available to authorized users.

## Background

The importance of dementia, a condition of memory and intellectual impairment, is increasing along with the increase in the older population. The total number of people with dementia worldwide in 2010 was estimated at 35.6 million and is projected to nearly double every 20 years to 65.7 million in 2030 and 115.4 million in 2050. This condition produces a great impact on the healthcare budget and social care [1]. Therefore, it has gained much attention.

Dementia, especially age-related dementia, is associated with many factors including forebrain and hippocampal atrophy [2, 3], acetylcholine (ACh) reduction [4], cholinergic hypofunction [5, 6], basal forebrain cholinergic neuron degeneration, neurotrophic signaling reduction [6] and excess oxidative stress [7]. Based on the crucial role of hypocholinergic function on dementia mentioned earlier, current anti-dementia drugs are targeted at the enhancement of cholinergic function. However, the current therapeutic efficacy is still limited, and adverse effects are commonly experienced [8]. Therefore, protection from dementia is required.

Medicinal plants have long been used for longevity promotion, neuroprotection and memory enhancement in traditional folklore. Both *Cyperus rotundus*, a plant in the Cyperaceae family, and *Zingiber officinale*, a plant in the Zingiberaceae family, are both reputed to exhibit longevity promotion. The phytochemical constituents of *C. rotundus* and *Z. officinale* have been studied extensively. *C. rotundus* contains quercetin, kaempferol, alkaloids, flavonoids, tannins, starch, glycosides, chalcones, gallic acid and p-coumaric acid [9, 10]. *Z. officinale* includes gingerol, paradols, and shogaol [11, 12]. Scientific data have demonstrated that *C. rotundus* and *Z. officinale* possess antioxidant, acetylcholinesterase inhibitory (AChEI), neuroprotective and memory-enhancing effects [13–18]. Based on the crucial role of hypocholinergic function and oxidative stress in dementia, the beneficial effect of both plants in dementia is the focus of this study. To optimize the benefit of the plant extracts, the positive modulation effect from the interaction of both plants has gained attention. We hypothesized that the combination of the extracts from *C. rotundus and Z. officinale* (CP1) could protect against age-related dementia. To test this hypothesis, we aimed to determine the antioxidant and AChEI effects of CP1. In addition, an in vivo study was also carried out to determine the neuroprotective effect of CP1 against age-related dementia in an animal model induced by a cholinotoxin, AF64A.

## Methods

### Plant collection and extract preparation

The aerial part of *C. rotundus* and the rhizome of *Z. officinale* were harvested from Khon Kaen province, Thailand from September – November 2012. *C. rotundus* was authenticated by Associate Professor Panee Sirisa-ard, from the Faculty of Pharmacy, Chiang Mai University, Thailand (voucher specimen No. 023159), and *Z. officinale* was authenticated by the National Museum of THAI Traditional Medicine, Thailand (voucher specimen No. 0002402). The plant materials were prepared as 95% alcoholic extracts. The percent yield of the *C. rotundus* and *Z. officinale* extracts were 7.41% and 10.48%, respectively. Based on our pilot in vitro study, a 1:5 ratio of *C. rotundus* to *Z. officinale* was found to exhibit the highest potential to protect against neurodegeneration induced by oxidative stress and increased the levels of neurotransmitters such as acetylcholine and dopamine, which play important roles in learning and memory (see Additional file 1: Table S1). Therefore, this ratio was selected for developing a novel neuroprotectant “CP1”. To control the quality of the developed neuroprotectant, the finger print of CP1 and the concentrations of gingerol and quercetin, the major chemical constituents of *Z. officinale* and C*. rotundus* that were previously reported to produce neuroprotection and memory enhancement [19, 20], were analyzed using high-performance liquid chromatography. The HPLC-UV analysis indicated that CP1 comprises gingerol, quercetin and several other unidentified peaks (See Additional file 1: Figure S1 and S2). In addition, semi-quantitative analysis revealed that the concentration of gingerol and quercetin was 65 and 7 mg/mL, respectively. The combined extract was kept at -20 °C in a dark bottle until use.

### Determination of antioxidant activity

Radical scavenging activity of 2,2-diphenyl-1-picrylhydrazyl (DPPH) radical of the combined extract of *Z. officinale* and *C. rotundus* (CP1) was determined spectrophotometrically [21]. The principle of the assay is based on the color change of the DPPH solution from purple to yellow when the radical is quenched by the antioxidant. In brief, 2.96 mL of a 0.1 mM solution of DPPH in methanol was incubated with 40 μL of various concentrations of extract (1.0, 2.0, 5.0, 10.0, 20.0, 25.0 mg/mL) at room temperature for 30 min. The decrease in DPPH radicals was evaluated by the optical density measurement at 515 nm. The stable free radical scavenging capacity is presented as the percentage of inhibition of DPPH radicals calculated according to the following equation: % inhibition of DPPH = (Abs control-Abs sample/Abs control) × 100

### Determination of antioxidant activity by ferric reducing antioxidant power (FRAP)

The ferric reducing antioxidant power assay was performed according to the procedure previously described [22] with some modifications. Briefly, the working FRAP reagent was mixed with 25 mL of 300 mM acetate buffer (3.1 g C_2_H_3_NaO_2_ · 3H_2_O and 16 mL C_2_H_4_O_2_), pH 3.6, 2.5 mL of 10 mM tripyridyltriazine (TPTZ) solution in 40 mM HCl, and 2.5 mL of 20 mM FeCl_3_ · 6H_2_O solution. Then, 1.8 mL of the FRAP solution was mixed with the CP1 extract (10 μL) in 1 mL distilled water. The absorbance of the reaction mixture at 593 nm was measured spectrophotometrically after incubation at 37 °C for 10 min. The results were expressed as μM ascorbic acid/100 g fresh weight.

### Determination of acetylcholinesterase (AChE) inhibition

AChE inhibitory activity was measured by using Ellman's colorimetric method [23]. Briefly, in 96-well plates, 25 μL of 15 mM ATCI, 75 μL of 3 mM DTNB and 50 μL of 50 mM Tris–HCl, pH 8.0, containing 0.1% bovine serum albumin (BSA), and 25 μL of the tested phytochemicals were added. The absorbance was measured at 405 nm after a 5-min incubation at room temperature. Then, 25 μL of 0.22 U.ml^-1^ of AChE was added and incubated for 5 min at room temperature, and the absorbance was measured at 412 nm. Acetylcholinesterase (5–1,000 μM) was used as a reference standard. The percentage inhibition was calculated using the following equation: Inhibition (%) = 1 – (A_sample_/A_control_) × 100, where A_sample_ is the absorbance of the sample extracts, and A_control_ is the absorbance of the blank (50% aqueous methanol in buffer).

In addition to the in vitro assay of AChE mentioned earlier, we also determined AChE activity in the hippocampal homogenate. In brief, the hippocampus was isolated and homogenized in ice-cold 0.1 M phosphate-buffered saline (pH 8.0). The homogenate was centrifuged at 1,000 g for 10 min at 4 °C, and the supernatant was used as the source of the enzyme in the AChE assay. AChE activity in hippocampus was evaluated using Ellman's method with slight modifications [24].

### Animals

Eight-week-old male Wistar rats weighing 180-220 g were used as experimental animals. They were derived from the National Laboratory Animal Center, Salaya, Nakorn Pathom. They were housed 6 per cage, maintained in a 12: 12 light: dark cycle, and given a standard pellet diet and water ad libitum. The experiments were performed to minimize animal suffering, and the experimental protocols were approved by the Animal Ethics Committee of Khon Kaen University, based on the Ethics of Animal Experimentation of National Research Council of Thailand (Confirmation No. AEKKU 41/2554).

### AF64A preparation

The preparation of AF64A was performed according to the method described by Hanin. In brief, an aqueous solution of acetylethylcholine mustard HCl (Sigma–Aldrich Co., USA) was adjusted to pH 11.3 with NaOH and stirred for 30 min. Then, the pH of the solution was adjusted to pH 7.4 with the gradual addition of HCl and stirred for 60 min at room temperature. The amount of AF64A was then adjusted to 2 nmol/2 μL. Artificial cerebrospinal fluid (ACSF) or vehicle of AF64A was distilled water, which was prepared in the same manner as AF64A.

### Surgical procedures

Sodium pentobarbital (Jagsonpal Pharmaceuticals LTD, Haryana, India) at a dose of 60 mg/kg BW was administered to the animals via the intraperitoneal route to induce anesthesia. The memory deficit was induced by the bilateral intracerebroventricular (i.c.v.) injection of AF64A (2 nmol/2 μL, 2 μL/side). Burr holes were made in the skull according to the following stereotaxic coordinates; posterior 0.8 mm, lateral ±1.5 mm, and ventral (from dura) 3.6 mm. AF64A was perfused via a 30-gauge needle that was inserted through the burr holes, and the perfusion rate was 1.0 μL/min. After being left at the injection site for 5 min, the needle was slowly withdrawn. The animals were allowed to recover from anesthesia and then placed in their cages.

### Experimental protocol

All rats were randomly assigned to 7 groups as follows:

- Group I Vehicle + ACSF; rats were orally given propylene glycol, which served as the vehicle to suspend the combined extract of CP1, once daily for 14 days after the administration of ACSF.
- Group II Vehicle + AF64A; rats were orally treated with propylene glycol once daily for a period of 14 days after the administration of AF64A.
- Group III Donepezil + AF64A; the animals were orally treated with donepezil (Aricept) (1 mg/kg BW), a cholinesterase inhibitor that is widely used as a standard drug for dementia treatment [25], once daily for a period of 14 days after the administration of AF64A.
- Group IV Vitamin C + AF64A; the animals were orally treated with vitamin C (250 mg/kg BW), a standard antioxidant that was previously reported to enhance memory and to attenuate neurodegeneration [25], once daily for a period of 14 days after the administration of AF64A.
- Group V-VII CP1 + AF64A; rats were treated with CP1 at doses of 100, 200 and 300 mg.kg^-1^ BW for a period of 14 days after the administration of AF64A.

Rats in all groups were orally given the assigned substances for a period of 14 days after the bilateral intracerebroventricular administration of AF64A. A memory assessment was performed every 7 days throughout the 14-day study period, whereas the measurements of the malondialdehyde (MDA) level and the activity of superoxide dismutase (SOD), catalase (CAT), glutathione peroxidase (GSH-Px) and acetylcholinesterase (AChE) in the hippocampus were performed at the end of study. Moreover, the density of the surviving neurons in various subregions of the hippocampus, including CA1, CA2, CA3 and the dentate gyrus, was also determined.

### Determination of spatial memory

Spatial memory was evaluated using the Morris water maze test. Rats were subjected to a metal pool (170 cm in diameter × 58 cm height) filled with tap water (25 °C, 40 cm deep). This pool comprised 4 quadrants including a northeast, southeast, southwest, and northwest quadrant. The water surface was covered with non-toxic milk. The removable platform was immersed below the water level at the center of one quadrant. All rats were trained to memorize the location of the invisible platform by forming the association of their location and the location of the platform using external cues. The time that the animal took to reach the top of the hidden platform was recorded as the escape latency or acquisition time. To determine the capability of the animals to retrieve and retain information, the platform was removed 24 hr later, and the rats were re-exposed to the same condition, except that the platform was removed. The time that each animal spent in the region that previously contained the platform was recorded as the retention time.

### Determination of the density of surviving neurons in the hippocampus

#### Histological study

Following induction of anesthesia with sodium pentobarbital (60 mg/kg BW), brain fixation was carried out by transcardial perfusion with a fixative solution containing 4% paraformaldehyde in 0.1 M phosphate buffer, pH 7.3. After the perfusion, the brain was removed and stored overnight in the fixative solution that was used in the perfusion, infiltrated with 30% sucrose solution and kept at 4 ° C. The specimens were frozen rapidly, and 10-μM thick coronal sections were prepared using a cryostat. All sections were rinsed in phosphate buffer and placed on slides coated with a 0.01% aqueous solution of a high molecular weight poly L-lysine.

### Morphological analysis

Five coronal sections from each rat in each group were studied quantitatively. The evaluation of the neuronal density in the hippocampus was performed under a light microscope at 40x magnification. The observer was blind to the treatment at the time of analysis. Viable stained neurons were identified on the basis of a stained soma with at least two visible processes. Counts were made in five adjacent fields, and the mean number was calculated and expressed as density of neurons per 255 μm^2^.

### Determination of oxidative stress markers

Rats were perfused with a cold saline solution to get rid of the blood from the brain tissue. Then, the hippocampus was isolated and prepared as a hippocampal homogenate, and the determination of the oxidative stress markers was performed. The malondialdehyde (MDA) level was indirectly estimated by determining the accumulation of thiobarbituric acid reactive substances (TBARS) [26]. To determine the activity of antioxidant enzymes, including superoxide dismutase (SOD), catalase (CAT) and glutathione peroxidase (GSH-Px), the hippocampus of each rat was weighed and homogenized with a buffer consisting of 10 mM sucrose, 10 mM Tris–HCl and 0.1 mM EDTA (pH 7.4). Then, a hippocampal homogenate was centrifuged at 3000 g at 4 °C for 15 min. The supernatant was separated and used for bioassays. The activity of SOD was determined using a xanthine/xanthine oxidase system as the source of superoxide radical production and the subsequent measurement of cytochrome *c* as a scavenger of the radicals. Optical density was measured using a spectrometer (UV-1601, Shimadzu) at 550 nm [27]. SOD activity was presented as units per milligram of protein (U mg^-1^ protein). One unit of enzyme activity was defined as the quantity of SOD required to inhibit the reduction rate of cytochrome *c* by 50%. CAT activity in the supernatant was measured by recording the reduction rate of H_2_O_2_ absorbance at 240 nm [28]. The activity of CAT was expressed as μmol H_2_O_2_.min^-1^mg^-1^ protein. GSH-Px was determined using *t*-butyl hydroperoxide as a substrate. The optical density was spectrophotometrically recorded at 340 nm and expressed as U mg^-1^protein [29]. One unit of the enzyme was defined as one micromole (μmol) of reduced nicotinamide adenine dinucleotide phosphate (NADPH) oxidized per minute_._

### Western blot analysis

The hippocampus was removed and rapidly frozen at -80 °C. The frozen tissue samples were homogenized in ice-cold RIPA buffer with protease inhibitors. The dissolved proteins were collected after centrifugation at 10,000 g for 30 min, and the supernatant was then collected. Protein concentration was determined using the NANOdrop Spectrophotometers. Equal amounts of protein (35 μg) were separated by SDS-PAGE (10% SDS-polyacrylamide gel electrophoresis) and transferred to a polyvinylidene difluoride (PVDF) membrane (Bio-Rad Laboratories, Hercules, CA). After transferring to the membrane, the blots were incubated in a blocking buffer (5% skim milk in Tris-buffer saline with 0.05% Tween-20) for 1 hr at room temperature and incubated overnight with antibodies against either phospho-ERK1/2 (1:1,000, Cell Signaling Cell Signaling Technology, Inc., Boston, MA, USA) or total ERK1/2 (1:1,000, Cell Signaling Cell Signaling Technology, Inc., Boston, MA, USA). After incubation, the membrane was subjected to several washing steps. An HRP-linked secondary antibody (1:2,000) was incubated with the membrane for 1 hr at room temperature, and signals were visualized by chemiluminescence using an ECL kit (Pierce, ThermoScientific). Images were evaluated by ImageQuant LAS 4000, GE Healthcare. Band densities were quantified with ImageQuant TL (IQTL) software, GE healthcare [30].

### Statistical analysis

Data were expressed as the means ± S.E.M. and analyzed statistically by one-way ANOVA, followed by a post hoc (LSD) test. The results were considered statistically significant at a *p*-value < 0.05.

## Results

### Antioxidant activity and acetylcholinesterase (AChE) inhibition of CP1

In the first part of this study, we determined and compared the antioxidant effect of *C. rotundus*, *Z. officinale* and the combined extract of *C. rotundus* and *Z. officinale* (CP1) by using DPPH and FRAP assays. In addition, acetylcholinesterase (AChE) inhibition was also determined using Ellman's colorimetric method. The results are shown in Table 1. Interestingly, our data clearly demonstrated that the combination of the *C. rotundus* and *Z. officinale* extracts (CP1) had a lower IC_50_ of FRAP (1.743 ± 0.003 mg/ml), DPPH (1.008 ± 0.001 mg/ml) and AChEI (0.100 ± 0.103 mg/ml) than those of the *C. rotundus* or *Z. officinale* extracts.

**Table 1:** FRAP, DPPH and AChEI activities of *Zingiber officinale*, *Cyperus rotundus* and CP1

Tested substance	FRAP IC_50_ mg/ml	DPPH IC_50_ mg/ml	AChEI IC_50_ mg/ml
*Zingiber officinale*	6.724 ± 0.005	2.086 ± 0.002	2.422 ± 0.133
*Cyperus rotundus*	8.822 ± 0.004	1.041 ± 0.001	0.382 ± 0.104
CP1	1.743 ± 0.003	1.008 ± 0.001	0.100 ± 0.103

### Effect of CP1 on spatial memory

**Fig. 1:**
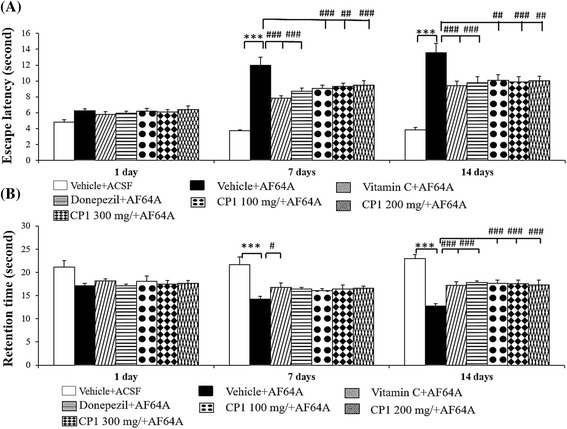
The effect of CP1, the combination extract of *C.rotundus* and *Z.officinale*, on spatial memory **a** effect of CP1 on escape latency **b** effect of CP1 on retention time (*n* = 8/ group) ***p-value < .001; compared to vehicle plus ACSF group; ^#,##,###^ *p*-value < .05, .01 and .001 respectively; compared to vehicle plus AF64A group

In this part, we mimicked the memory impairment condition observed in age-related dementia in humans by inducing a hypocholinergic condition via the bilateral administration of AF64A, a cholinotoxin, into the lateral ventricles. Figure 1a and b showed that vehicle + ACSF showed no significant changes in both escape latency and retention time. Our data showed that the administration of AF64A significantly enhanced escape latency (*p*-value < .001 for all compared to the vehicle + ACSF group) but decreased the retention time (*p*-value < .001 compared to the vehicle + ACSF group) on both the 7th and 14th day. Both donepezil and vitamin C treatments significantly mitigated the enhanced escape latency induced by AF64A (*p*-value < .001 compared to the vehicle + AF64A group). Donepezil also mitigated the decreased retention time induced by AF64A both at 7 and 14 days of treatment (*p*-value < .05 and .001, respectively, compared to the vehicle + ACSF group). Ascorbic acid only mitigated the decreased retention time at 14 days of treatment (*p*-value < .001 compared to the vehicle + ACSF group). Interestingly, all doses of CP1 (100, 200 and 300 mg.kg^-1^) significantly mitigated the enhanced escape latency at 7 (*p*-value < .001, .01 and .001, respectively, compared to the vehicle + AF64A group) and 14 days of treatment (*p*-value < .01, .001 and .01, respectively, compared to the vehicle + AF64A group). In addition, CP1 at all doses used in this study also mitigated the decreased retention time induced by AF64A at 14 days of treatment (*p*-value < .001 for all compared to the vehicle + AF64A group).

### Effect of CP1 on hippocampal neurodegeneration

**Fig. 2:**
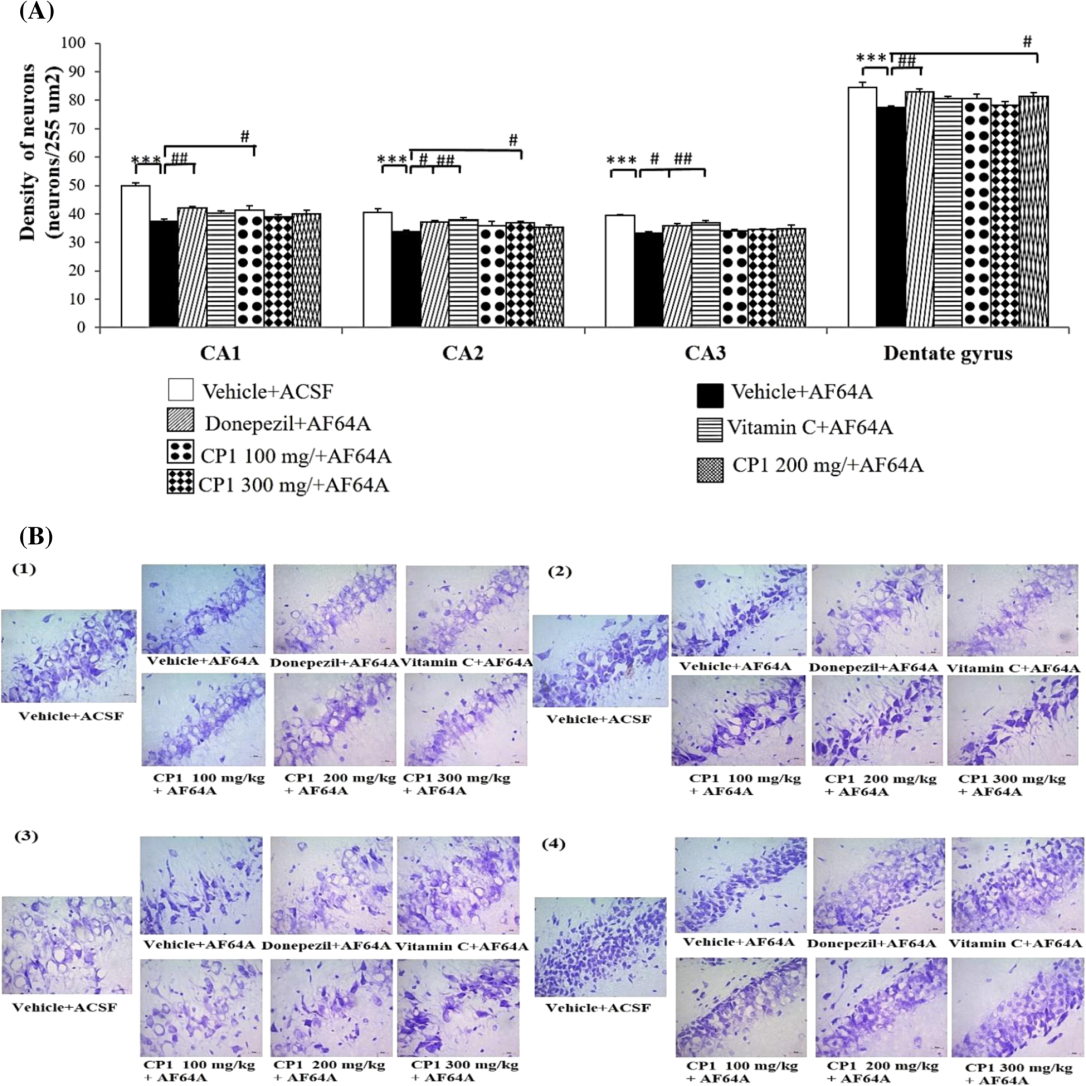
The effect of CP1 on neuron density in various subregions of hippocampus including CA1, CA2, CA3 and dentate gyrus. **a** Average density of neurons in CA1, CA2, CA3 and dentate gyrus **b** Photograph of neuron density in (1) CA1, (2) CA2, (3) CA3 and (4) dentate gyrus. (n = 8/group) ^***^ *p*-value < .001; compared to vehicle + ACSF group. ^#, ##, ###^ *p*-value < .05, .01 and .001 respectively; compared to vehicle + AF64A group

Figure 2 (see Additional file 1: Figure S3) shows the effect of CP1 on neuronal density in the hippocampus. The results showed that AF64A significantly decreased neuronal density in the CA1, CA2, CA3 and dentate gyrus (*p*-value < .001 for all compared to the vehicle + ACSF group). Rats subjected to AF64A that received donepezil showed a significant elevation in neuronal density only in the CA1, CA2, CA3 and dentate gyrus (*p*-value < .01, .05, .05, .01, respectively, compared to the vehicle + AF64A group). In addition, vitamin C significantly enhanced the neuronal density in the CA2 and CA3 in rats subjected to AF64A treatment (*p*-value < .01 and .01, respectively, compared to the vehicle + AF64A group). Interestingly, CP1 at a low concentration (100 mg/kg) significantly attenuated the reduction in the neuronal density in the CA1 (*p*-value < .05 compared to the vehicle + AF64A group) in rats that received AF64A. An enhanced neuronal density in the CA2 and dentate gyrus was observed in rats subjected to AF64A that received CP1 at doses of 200 and 300 mg.kg^-1^ BW (*p*-value < .05 compared to the vehicle + AF64A group). No significant changes were observed in the CA3.

### Effect of CP1 on oxidative stress markers

The effects of CP1 on oxidative stress markers, including the level of MDA and the activity of SOD, CAT and GSH-Px in the hippocampus, were also evaluated. The results are shown in Table 2. AF64A injection was demonstrated to significantly increase the MDA level (*p* < .001 compared to the vehicle + ACSF group) but decrease the activity of SOD, CAT and GSH-Px (*p* < .001 for all compared to the vehicle + ACSF group). The elevation in the MDA level in the hippocampus induced by AF64A was mitigated by donepezil, vitamin C and all doses of CP1 (*p*-value < .01, .05, .05, .01 and .05, respectively, compared to the vehicle + AF64A group). Increased CAT activity was also observed in rats subjected to AF64A that received donepezil, vitamin C and all doses of CP1 (*p*-value < .01, .01, .05, .01. 01, respectively, compared to the vehicle + AF64A group). All treatments mentioned earlier failed to modulate the reduction in GSH-Px induced by AF64A.

**Table 2:** Effect of CP1 on the oxidative stress markers in hippocampus

Group	MDA (nmol/mg · protein)	SOD (unit/mg · protein)	GSH-Px (unit/mg · protein)	CAT (unit/mg · protein)
Vehicle + ACSF	0.0008 ± 0.0002^###^	3.3230 ± 0.1195^###^	0.0829 ± 0.0155	1 09.2071 ± 1.5467^###^
Vehicle + AF64A	0.0021 ± 0.0005^***^	2.1633 ± 0.1244^***^	0.0518 ± 0.0163	84.6617 ± 2.5905^***^
Donepezil + AF64A	0.0011 ± 0.0001^##^	2.9833 ± 0.3891^##^	0.0743 ± 0.0185	106.4200 ± 5.6561^##^
Vitamin C + AF64A	0.0011 ± 0.0001^#^	3.1532 ± 0.1500^###^	0.0870 ± 0.0095	106.8167 ± 3.6787^##^
CP1 100 mg/kgBW + AF64A	0.0013 ± 0.0002^#^	2.9210 ± 0.2465^##^	0.0705 ± 0.0235	101.3417 ± 6.1163^#^
CP1 200 mg/kgBW + AF64A	0.0011 ± 0.0001^##^	2.4226 ± 0.0834^***^	0.0624 ± 0.0096	105.8271 ± 3.6703^##^
CP1 300 mg/kgBW + AF64A	0.0015 ± 0.0001^#^	2.3510 ± 0.0531^***^	0.0612 ± 0.0088	102.3483 ± 5.4788^##^

^*, ***^ *p*-value < .05 and.001 respectively; compared to vehicle + ACSF group. ^#, ##, ###^ *p*-value < .05, .01 and .001 respectively; compared to vehcle + AF64A group)

### Effect of CP1 on acetylcholinesterase (AChE) activity

**Fig. 3:**
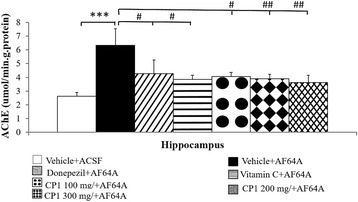
Effect of CP1 on an acetylcholinesterase (AChE) enzyme activity in hippocampus. (n = 8/group) ^***^ *p*-value < .001; compared to vehicle + ACSF group. ^#, ##^ *p*-value < .05 and .01 respectively; compared to vehicle + AF64A group

The effect of CP1 on cholinergic function was evaluated indirectly by using the activity of AChE as an indirect indicator reflecting the available acetylcholine in the hippocampus. The results are shown in Fig. 3. Rats exposed to AF64A showed an elevation in AChE (*p*-value < .001 compared to the vehicle + ACSF group). However, this change was reversed by donepezil, vitamin C and all doses of CP1 (*p*-value < .05, .05, .05, .01 and .01, respectively, compared to the vehicle + AF64A group).

### Effect of CP1 on ERK1/2 activation

**Fig. 4:**
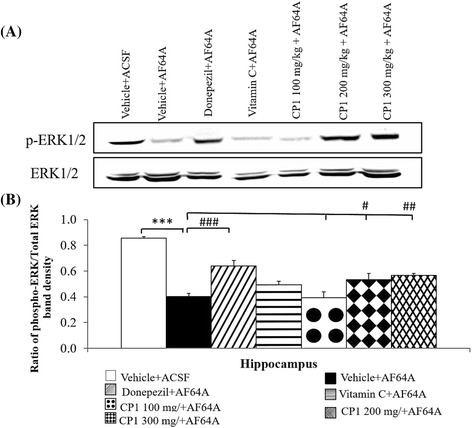
Effect of CP1 on the level of ERK1/2 and pERK1/2 in hippocampus. **a** Western blots for phospho-(p)-ERK1/2 and total ERK1/2 **b** the ratio of phospho-ERK to total ERK band densities (n = 8/group). ^***^ *p*-value < .001; compared to vehicle + ACSF group. ^#, ##, ###^ *p*-value < .05, .01 and .001 respectively; compared to vehicle + AF64A group

Since the ERK cascade plays an important role in synaptic plasticity, long-term potentiation and cell survival, the effect of CP1 on ERK1/2 in the hippocampus was also assessed. The results are shown in Fig. 4. AF64A injection was found to significantly decrease phosphorylation of ERK1/2 (p-value < .001 compared to the vehicle + ACSF group). Interestingly, enhanced phosphorylation of ERK1/2 was observed in the AF64A-treated rats that received donepezil and those that received medium and high doses of CP1 (*p*-value < .001, .05 and .01, respectively, compared to the vehicle + AF64A group). No significant change was observed in the AF64A-treated rats that received either vitamin C or low doses of CP1.

**Fig. 5:**
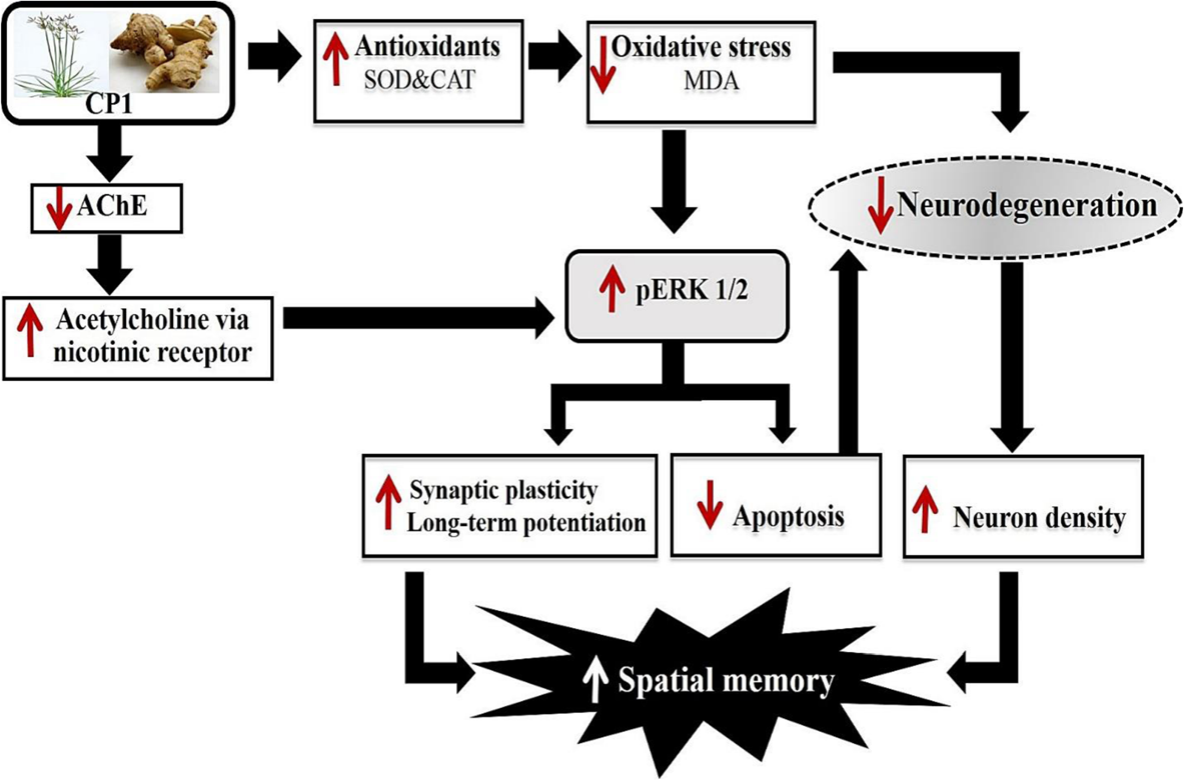
Schematic diagram shows the possible underlying mechanism of CP1

## Discussion

Medicinal plants have long been used for treating various ailments either as single plants or as polyherbal recipes. However, the polyherbal recipes have been more widely used than the single plants based on the concept that the synergistic effect of multiple plants can provide more beneficial effects [31]. However, less scientific evidence is available. In this study, we have clearly demonstrated that CP1, the combined extract of *C. rotundus* and *Z. officinale*, showed a lower IC_50_ of both the antioxidant effect via DPPH and the AChEI effect. Therefore, our results confirmed the hypothesis that the interaction of both medicinal plants mentioned earlier could provide a greater benefit. This was also in agreement with other studies that have demonstrated the beneficial effect of the combined extract [32–34].

The current results also demonstrated that CP1 significantly increased spatial memory, enhanced cholinergic function and decreased oxidative stress in the hippocampus. The current data revealed that CP1 at all doses in this study increased CAT activity, and the low dose of CP1 increased SOD activity. Therefore, the increase in CAT activity with SOD activity might involve the reduction of oxidative damage [35].

In addition, CP1 also significantly enhanced the density of neurons in the CA1, CA2 and dentate gyrus and increased pERK1/2 levels in these same areas. ERK1/2, a subclass of mitogen-activated protein (MAP) kinases, has been reported to play a pivotal role in neurodegeneration via the mitochondrial apoptotic mechanism [36–38]. Neurodegeneration in the hippocampus, an important area for learning and memory, is associated with memory deficits [39, 40]. Therefore, the memory-enhancing effect of CP1 may occur partly via decreased oxidative stress by enhancing the activity of antioxidant enzymes in the hippocampus, which, in turn, could induce an increase in pERK1/2 [41], giving rise to an increased neuronal density in the CA1, CA2 and dentate gyrus, leading to improvements in the encoding, retrieval and consolidation processes resulting in enhanced spatial memory [25]. Although the decreased oxidative stress could increase the phosphorylation of ERK1/2, resulting in an anti-apoptotic effect and leading to enhanced neuronal density in the hippocampus, no close relationship between the increase in pERK1/2 and the decrease in oxidative stress was observed, especially at the low concentration of CP1. Since decreased oxidative stress in rats with AF64A–induced memory deficits can increase the neuronal density in the hippocampus and can improve memory impairment [42, 43], we suggested that the antioxidant effect of CP1 might decrease oxidative stress status in the hippocampus, which in turn would decrease neurodegeneration induced by the attack of free radicals, resulting in increased neuronal density in this area. In addition, the activation of ERK1/2 gives rise to the phosphorylation of ERK1/2, which in turn plays an important role in the function of acetylcholine via the nicotinic receptor [44]. Therefore, it is also possible that CP1 at all doses used in this study may suppress AChE, leading to an increase in the available acetylcholine (ACh), which, in turn, may bind to the nicotinic receptor, resulting in the activation and phosphorylation of ERK1/2 and finally leading to improved spatial memory. These effects have been shown in Fig. 5.

Our results also showed differential vulnerability to CP1. The CA3 region showed less vulnerability among the various subregions assessed in this study. The possible explanation may be due to differences in the distribution of signal molecules and growth factors that play important roles in cell survival [44].

Our data failed to show dose-dependent effects. The possible explanation might be related to the masking effect of non-active ingredients. In addition, the relationship between the concentration of CP1 and the observed parameters might not be a simple linear relationship, and the active ingredient may also exert the beneficial effect indirectly via other signal transduction process such as ERK1/2. Since no significant differences among doses were observed, we suggested that the medium dose would be the most appropriate dose for application based on its benefit in all parameters, including the effect on the ERK signal pathway. Since this dose could effectively exert a positive modulation effect on multiple targets, it could also provide a greater benefit. In addition, the medium dose also provides a lower risk for toxicity than the high dose of CP1.

A limitation of this study is that all ingredients of the combined extract are not determined. Based on previous studies, it has been demonstrated that gingerol [45] and quercetin [46] exert protective effects against oxidative stress-related neurodegeneration. Therefore, we measured the concentrations of the mentioned substances in the combined extract. Since both substances were also found in the combined extract, and the observed effect was similar to the effect of both substances, we suggested that they might be partly responsible for the neuroprotective effect of CP1 in this study. In addition to the direct effect of both substances mentioned earlier, interaction effects of various ingredients, including the interaction of both ingredients and the effect of other constituents, are still possible. However, further investigations are necessary to provide better understanding concerning the possible active ingredients.

## Conclusion

CP1, the combined extract of *C. rotundus* and *Z. officinale*, is a potential supplement to improve neurodegeneration and memory impairment. The possible mechanism for its beneficial effects may be through improving oxidative stress status, which in turn would increase pERK1/2 in the hippocampus, leading to improvement in memory impairment. In addition, CP1 can also suppress AChE activity in the hippocampus, giving rise to increased available ACh and increased function of ACh via the nicotinic receptor, resulting in enhanced memory performance. However, further studies are necessary to investigate the precise active ingredients and subchronic toxicity of CP1 and its interaction with drugs that are commonly used in elderly patients to assure safe consumption.

## Additional file

Additional file 1: The pilot in vitro study results, the experimental details for the HPLC-UV analysis and UV absorption of CP1 and photograph of neuron density in hippocampus. (DOCX 874 kb)

## Abbreviations

Ach: Acetylcholine
AChE: Acetylcholinesterase
ACSF: Artificial cerebrospinal fluid
AD: Alzheimer’s disease
AF64A: Ethylcholine mustard aziridinium
CA1: Cornu ammonis area 1
CA2: Cornu ammonis area 2
CA3: Cornu ammonis area 3
CAT: Catalase
DG: Dentate gyrus
DPPH: 2,2-diphenyl-1-picrylhydrazyl
ERK: Extracellular signal-regulated kinase
FRAP: Ferric reducing antioxidant power
GSH-Px: Glutathione peroxidase
IC_50_: Median Inhibition Concentration
MAPK: Mitogen-activated protein kinases
MDA: Malondialdehyde
pERK1/2: Phosphorylated extracellular signal-regulated kinase 1 and 2
SOD: Superoxide dismutase
TPTZ: tripyridyltriazine
