# Supplementary material for: Neuroprotective and cognitive-enhancing effects of the combined extract of Cyperus rotundus and Zingiber officinale
Source: BMC Complement Altern Med. 2017 Mar 3;17:135. doi: 10.1186/s12906-017-1632-4 (PMC5335841; doi:10.1186/s12906-017-1632-4)
Supplement: Additional file 1: — The pilot in vitro study results, the experimental details for the HPLC-UV analysis and UV absorption of CP1 and photograph of neuron density in hippocampus. (DOCX 874 kb) [file 12906_2017_1632_MOESM1_ESM.docx]

**Additional File 1**

**Table S1.** The total phenolic compounds, antioxidant activity and the inhibitory activities of AChE and MAOB of various combinations of *C. rotundus* and *Z. officinale*

| ***C. rotundus : Z. officinale*** | **Total phenolics** | **FRAP IC_50_** | **DPPH IC_50_** | **AChEI IC_50_** | **MAOBI IC_50_** |
| --- | --- | --- | --- | --- | --- |
|  | mg GAE/100 g plant extract | mg/ml | mg/ml | mg/ml | mg/ml |
| 0:1 | 131.371±0.003 | 6.724 | 2.086 | 2.422 | >5 |
|  |  |  |  |  |  |
| 1:0 | 180.133±0.003 | 8.822 | 1.041 | 0.382 | >5 |
|  |  |  |  |  |  |
| 1:1 | 173.562±0.009 | 3.429 | 1.35 | <0.1 | >5 |
|  |  |  |  |  |  |
| 1:2 | 216.371±0.018 | 3.293 | 1.181 | <0.1 | 2.078 |
|  |  |  |  |  |  |
| 1:3 | 219.657±0.009 | 2.889 | 1.458 | >5 | 0.839 |
|  |  |  |  |  |  |
| 1:4 | 212.990±0.001 | 4.254 | 1.426 | 0.197 | 1.594 |
|  |  |  |  |  |  |
| 1:5 | 258.467±0.002 | 1.743 | 1.008 | <0.1 | <0.1 |
|  |  |  |  |  |  |
| 2:1 | 240.514±0.003 | 3.026 | 1.169 |  | <0.1 |
|  |  |  |  | <0.1 |  |
| 2:3 | 233.133±0.010 | 1.962 | 1.489 | <0.1 | <0.1 |
|  |  |  |  |  |  |
| 2:5 | 237.229±0.002 | 7.873 | 1.345 | <0.1 | <0.1 |
|  |  |  |  |  |  |
| 3:1 | 205.276±0.004 | 3.908 | 3.565 | <0.1 | <0.1 |
|  |  |  |  |  |  |
| 3:2 | 214.371±0.002 | 2.711 | 1.787 | <0.1 | <0.1 |
|  |  |  |  |  |  |
| 3:4 | 236.705±0.006 | 1.981 | 1.624 | 0.475 | <0.1 |
|  |  |  |  |  |  |
| 3:5 | 223.657±0.001 | 2.574 | 1.883 | <0.1 | <0.1 |
|  |  |  |  |  |  |
| 4:1 | 240.800±0.009 | 2.392 | 1.486 | <0.1 | 0.448 |
|  |  |  |  |  |  |
| 4:3 | 244.848±0.003 | 1.807 | 1.296 | <0.1 | <0.1 |
|  |  |  |  |  |  |
| 4:5 | 246.705±0.005 | 1.746 | 1.027 | <0.1 | <0.1 |
|  |  |  |  |  |  |
| 5:1 | 235.514±0.003 | 2.769 | 0.906 | <0.1 | <0.1 |
|  |  |  |  |  |  |
| 5:2 | 239.324±0.001 | 2.273 | 0.854 |  |  |
|  |  |  |  | >5 | <0.1 |
| 5:3 | 232.705±0.008 | 2.836 | 0.84 | >5 | <0.1 |
|  |  |  |  |  |  |
| 5:4 | 243.800±0.003 | 10.232 | 0.805 | >5 | <0.1 |
|  |  |  |  |  |  |

**HPLC-UV Analysis**

**Experimental**

HPLC-UV for the qualitative and semi-quantitative analysis of the gingerol and quercetin was performed on Water 515 HPLC ump (Waters corporation). The data were acquired and processed using Empower^TM^ 3 software. Separation of two constituents, gingerol and quercetin, was achieved on a Purospher®STAR, C18 column (250 mm × 4.6 mm, 5 μm, Sorbet Lot No. HX255346, Merck). The mobile phases consisted of 100%Methanol (A) and 2.5%Acetic acid (B). The gradient flow was as follows: 10 % A for 0 min, 70% A for 17 min, 100 % A for 18–20 min and 10 % A for 22–25 min. Re-equilibration time was 40 min. The flow rate was 1.0 mL/min and injection volume was 10-20 μL. Standard gingerol (5 mg/mL, Sigma–Aldrich Co., USA, 98% purity,) and quercetin (20 mg/mL, Sigma–Aldrich Co., USA, 98% purity) were prepared as a solution in DI water. The CP1 sample was prepared as the following: 200 mg of lyophilized sample was dissolved in 1 mL of distilled water and was centrifuged for 10 minutes. The solution was filtered through a 0.2 μm syringe filter (Pall Life Sciences, Ann Arbor, MI) before injection into the HPLC system.

**Results and discussion**

Gingerol and quercetin were determined qualitatively by HPLC-UV method. Figure S1 (A) showed the chromatogram for a CP1 sample that contains gingerol (3.647 min) and quercetin (19.484 min) and several other peaks detected at the wavelength of 270 nm. The retention time agrees well with the standard gingerol (Figure S2 (B), 3.518 min) and quercetin (Figure S1 (C), 19.560 min). Further analysis of the UV absorption in the range of 200-400 nm of gingerol (Figure S2 (A)) and quercetin (Figure S2 (B)) demonstrated that the peaks correspond well to the spectra reported in the literature [1,2] for standard gingerol and quercetin, respectively. However, we are not able to determine other chemical components that appear in chromatogram due to the complexity nature of the crude extract. Also, the semi-quantitative analysis revealed that the concentration of gingerol and quercetin are 65 and 7 mg/mL, respectively.


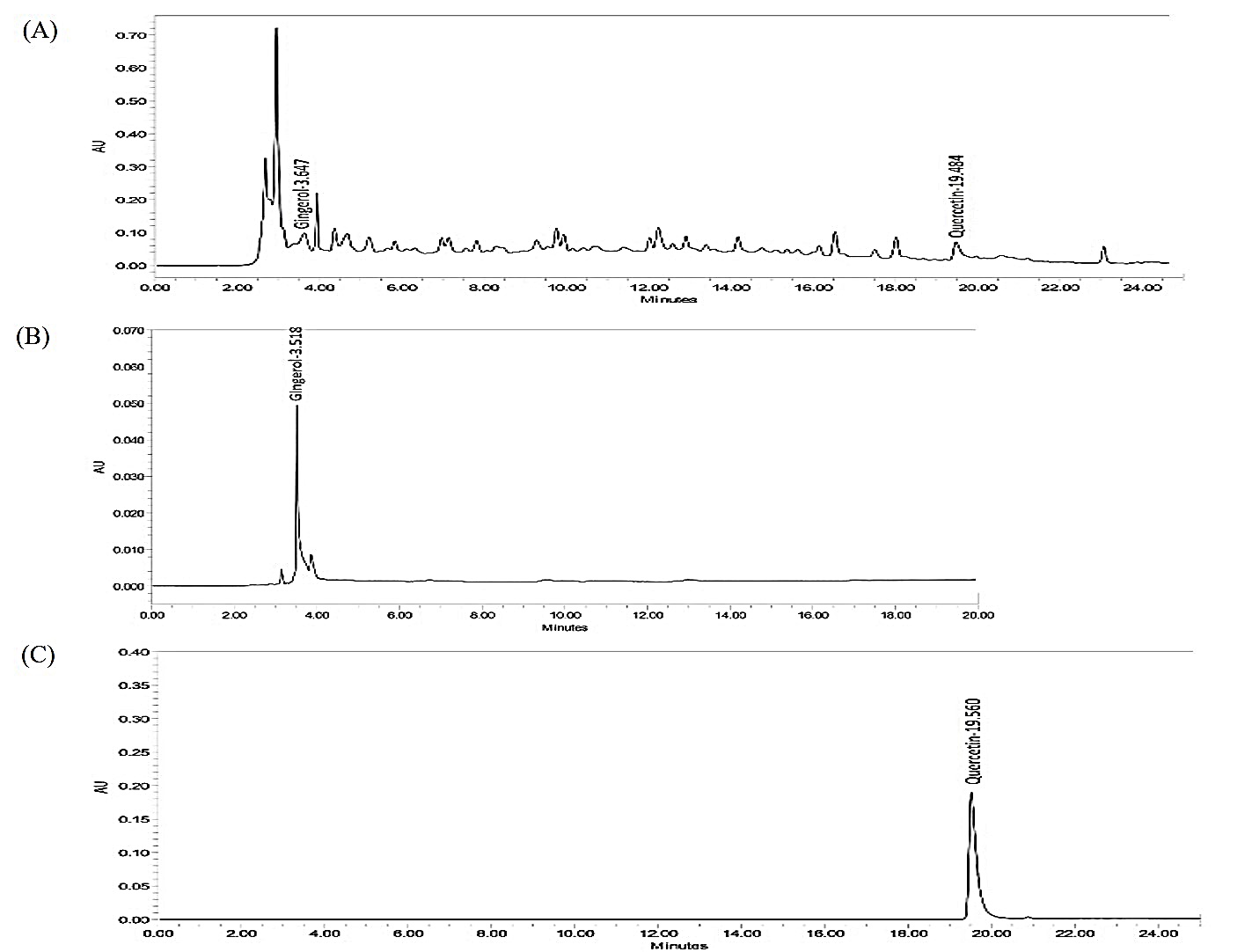


**Fig. S1** High performance liquid chromatography (HPLC) chromatogram of the combined extract of *Zingiber officinale* and *Cyperus rotundus* (A), standard of gingerol (B) and standard of quercetin (C).


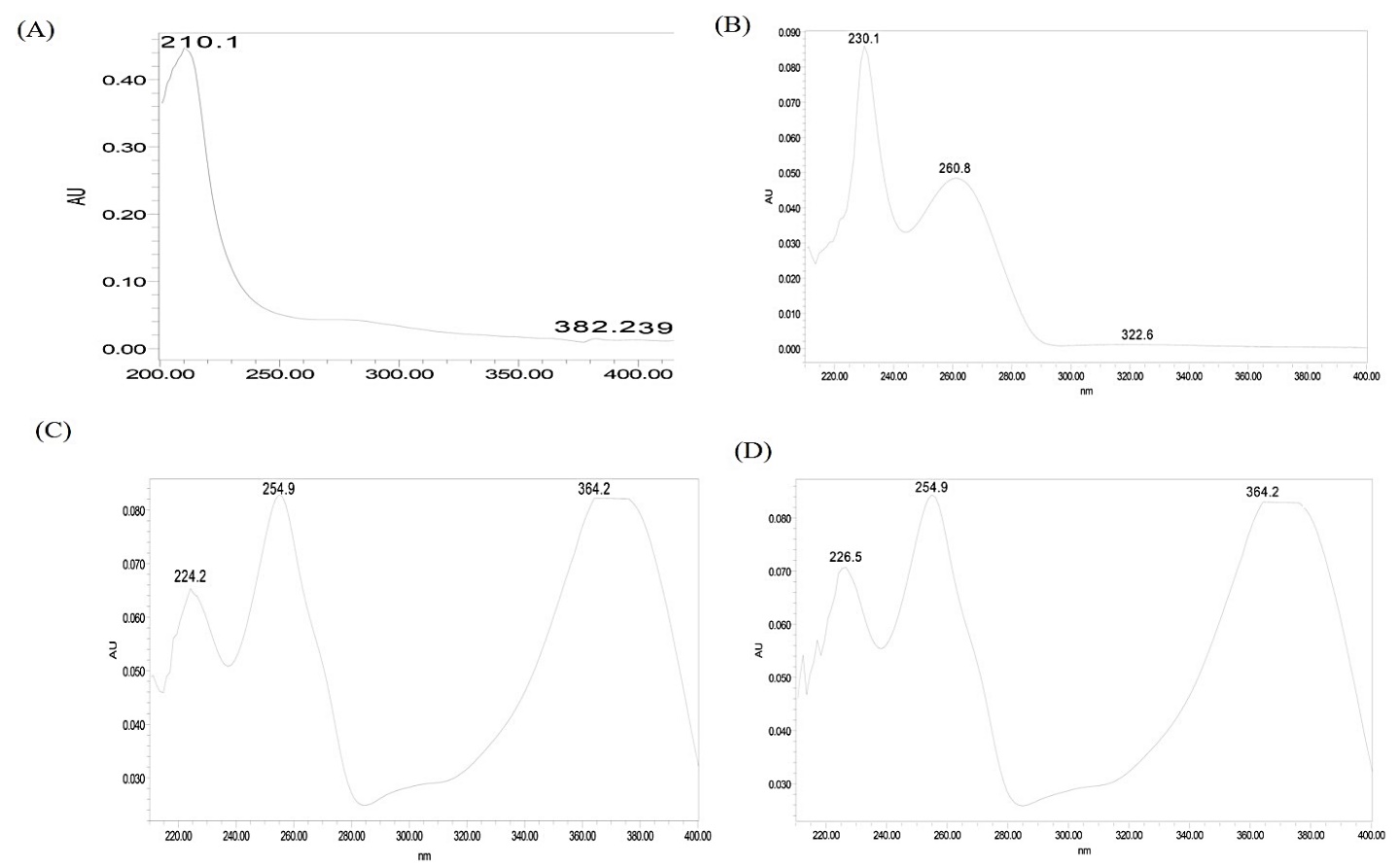


**Fig. S2** UV-vis spectra of the standard of gingerol (A), gingerol in CP1 (B), standard of quercetin (C) and quercetin in CP1 (D).

**References**

[1] Guo YH, Zhang Y, Luo D, Tao Ma. **Raw Ginger Composite Antioxidant with High Efficiency to Extract Vitamin C from Strawberry.** *Adv Chem Eng Sci*. 2013; **3**: 185-188.

[2] Duan Y. **Ultraviolet-Visible spectrum characterizations of Quercetin in aqueous ethanol solution with different pH values**. *J Chem Pharm Res*. 2014; **6(9)**: 236-240.


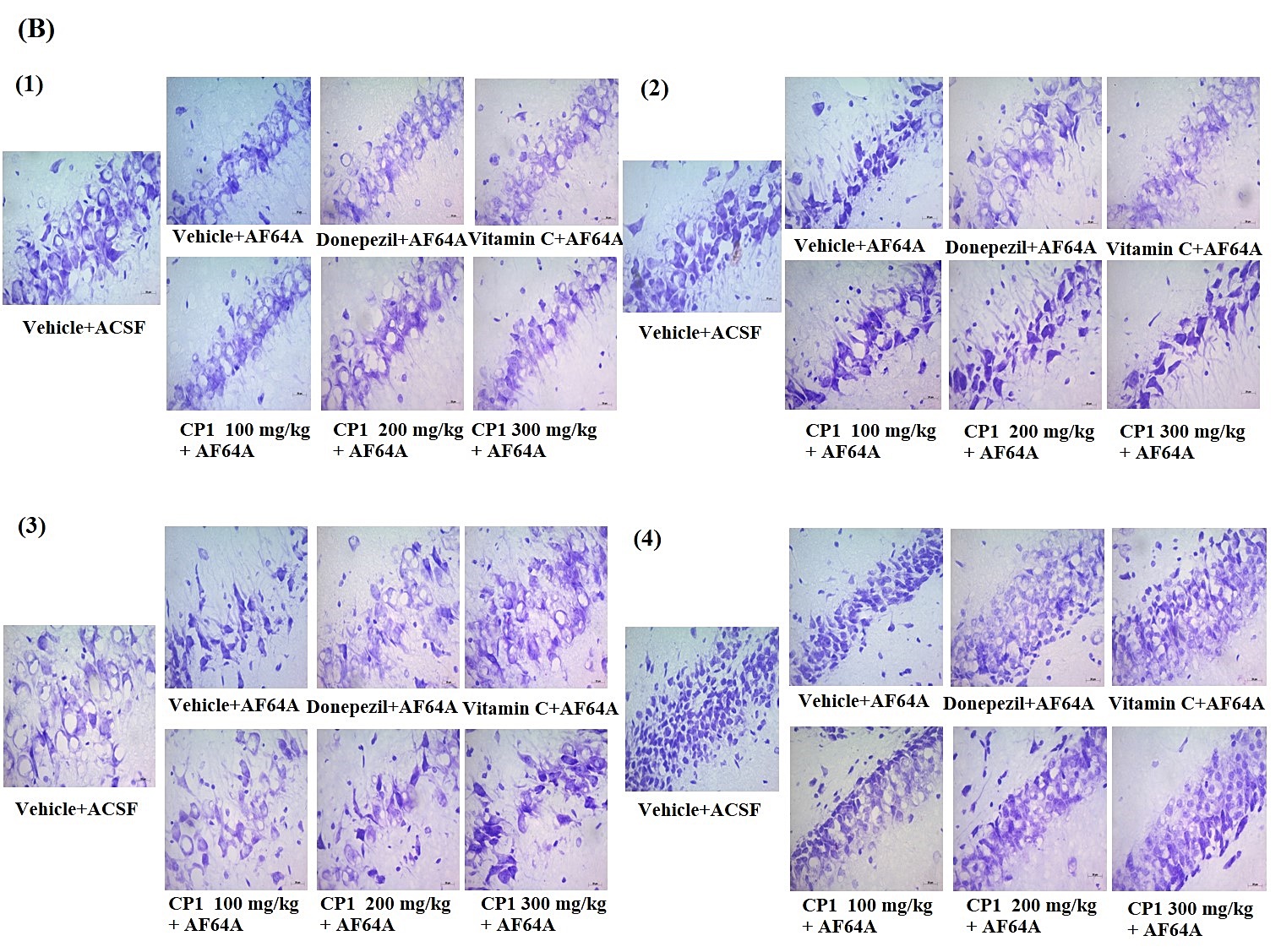


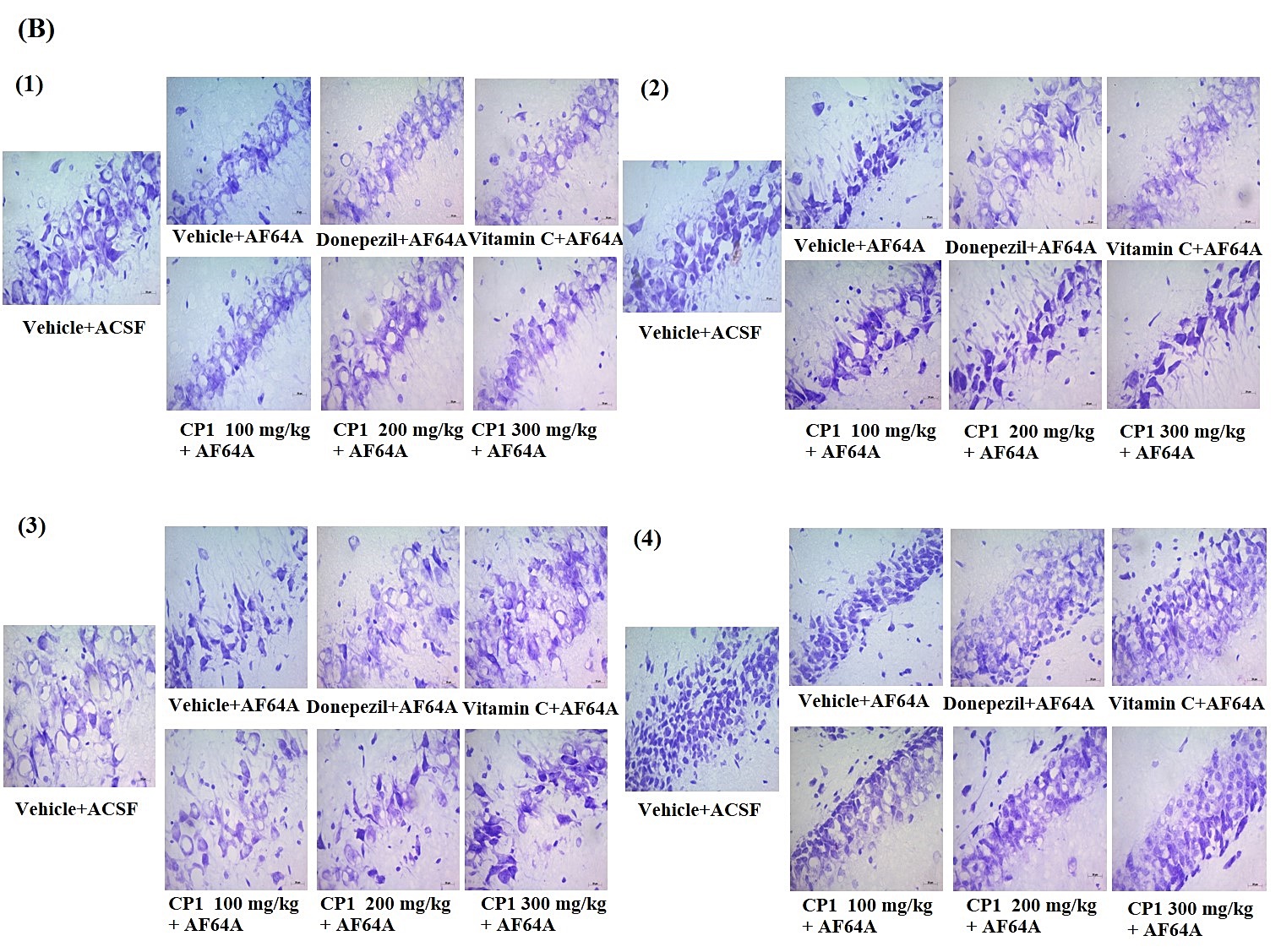


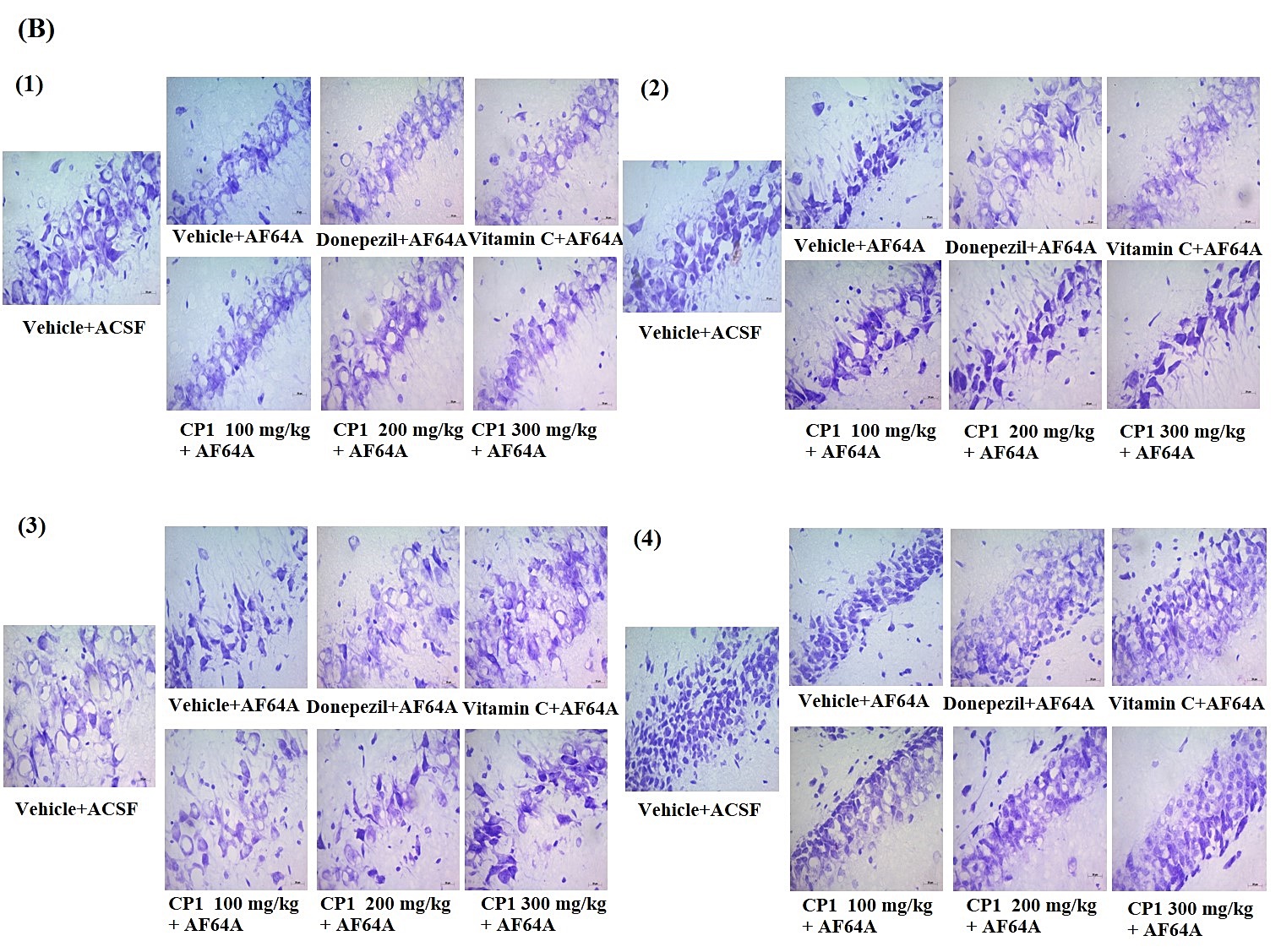


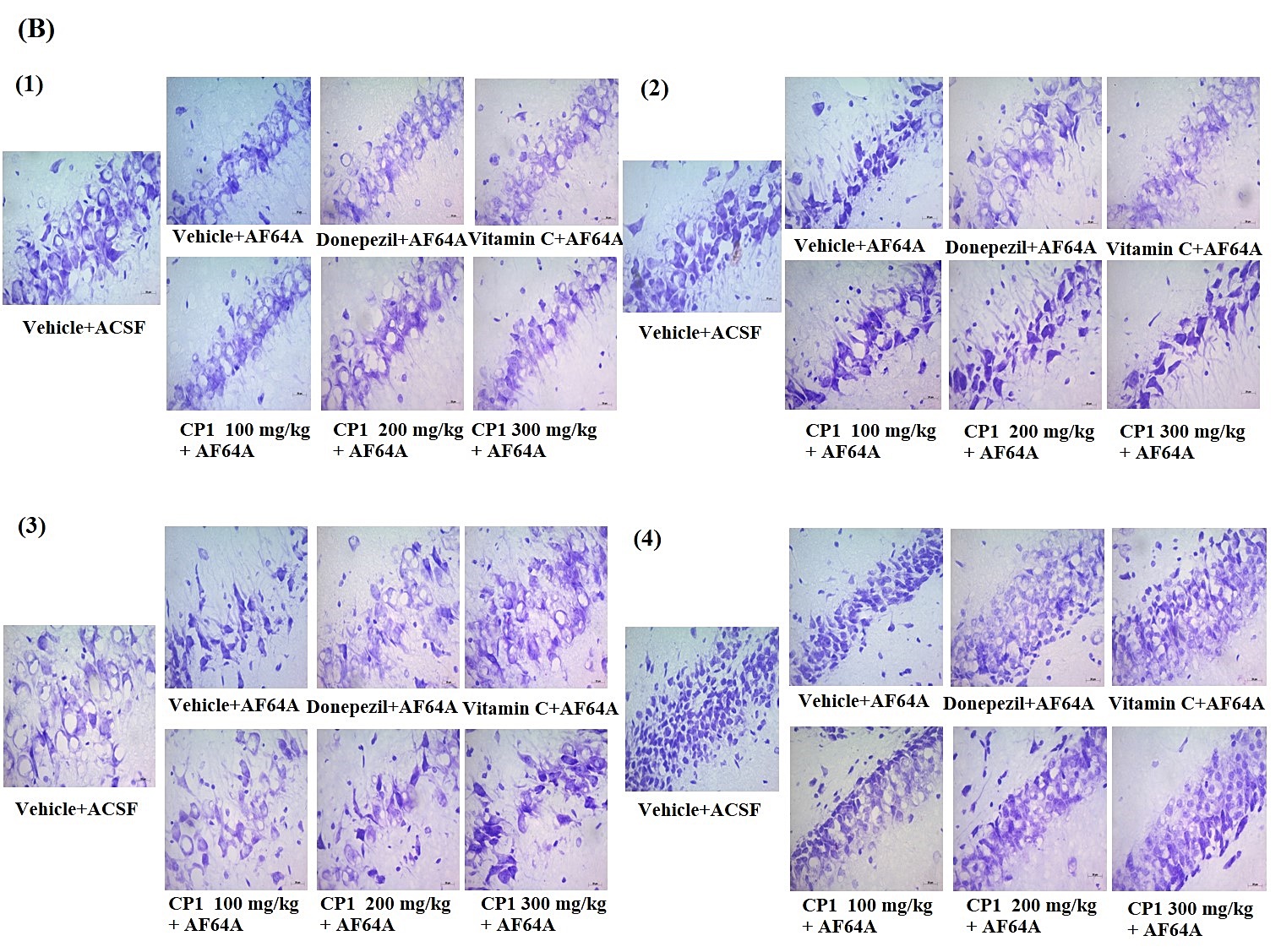


**Fig. S3** Photograph of neuron density in (1) CA1, (2) CA2, (3) CA3 and (4) dentate gyrus.
